# Modulation of toxicity effects of CuSO_4_ by sulfated polysaccharides extracted from brown algae (*Sargassum tenerrimum*) in *Danio rerio* as a model

**DOI:** 10.1038/s41598-023-38549-0

**Published:** 2023-07-15

**Authors:** Ashkan Zargari, Mohammad Nejatian, Sepideh Abbaszadeh, Kambiz Jahanbin, Tahereh Bagheri, Aliakbar Hedayati, Monireh Sheykhi

**Affiliations:** 1 Department of Nutrition Science and Food Hygiene, Faculty of Health, Baqiyatallah University of Medical Sciences, Tehran, Iran; 2 Health Research Center, Life Style Institute, Baqiyatallah University of Medical Sciences, Tehran, Iran; 3 Faculty of Agricultural Engineering, Department of Food Science and Technology, Shahrood University of Technology, Shahrood, Iran; 4 Offshore Water Research Center (OWRC), Iranian Fisheries Science Research Institute, Agricultural Research, Education and Extension Organization (AREEO), Chabahar, Iran; 5 Faculty of Fisheries and Environmental Sciences, Gorgan University of Agricultural Sciences and Natural Resources, Gorgan, Iran; 6 Student Research Committee, Baqiyatallah University of Medical Sciences, Tehran, Iran

**Keywords:** Immunology, Environmental sciences

## Abstract

Copper is widely used in agriculture and aquaculture due to its high disinfection properties and relatively low cost. However, the increase in copper concentration due to evaporation can lead to water reservoir pollution, which can harm the health of consumers. The present study aimed to determine the role of sulfated polysaccharides (SPs) extracted from *Sargassum tenerimum* algae in reducing lesions caused by the heavy metal copper. Zebrafish (*Danio rerio*) were used as a human model in five treatments. The negative and positive control groups were fed a diet containing zero percent of SPs, while the experimental groups were fed 0.5%, 1%, and 1.5% of SPs in three treatments for 56 days, finally CuSO_4_ was exposed only to the positive control group and the groups fed with SPs. Results showed a significant decrease in the activity level of ALT enzymes (39–16 U/mL), AST (67–46 U/mL), and ALP (485–237 U/mL), confirming the results obtained from histopathological studies in CuSO_4_ exposed groups. The addition of SPs to the diet resulted in a significant reduction (sig < 0.05) of mortalities due to the decrease of tissue damage. Additionally, due to the anti-inflammatory properties and the protective effect of SPs, a significant decrease (sig < 0.05) was observed in the relative expression of Il-1β and Tnf-α genes.

## Introduction

Copper compounds such as CuSO_4_ are commonly used as a method to control parasitic diseases caused by crustaceans, unwanted algae growth, and the growth of fungi and bacteria in water resources. Furthermore, this compound is used in the agricultural sector as a fungicide to control fungal and bacterial diseases in fruits and vegetables^1^. CuSO_4_ is also involved in the formation of chlorophyll and the phenomenon of photosynthesis, and stimulates the production of vitamin A, making it a useful fertilizer^2^. However, the excessive use of CuSO_4_ and the use of high concentrations in the agriculture and aquaculture sectors can contaminate water sources, which may be detrimental to the environment, consumption, and target consumers, despite its sterilizing properties and relatively low cost.

One effective method for controlling the effects of heavy metals can be through the use of natural bioactive compounds, which not only have the ability to enhance the body's immunity but can also reduce the harmful effects caused by heavy metals^2,3^. Seaweeds are a rich source of polysaccharides, especially in the cell wall structure, and are readily available and cost-effective sources for extracting sulfated polysaccharides (SPs) such as alginate and fucoidan. Polysaccharides are polymers of simple sugars linked together by glycosidic bonds and are widely used in various products, including stabilizers, emulsifiers, foods, and beverages^4^. Natural bioactive compounds found in algae, such as carrageenan, agar, alginate, fucoidan, mannitol, and others, possess chelating properties that enable them to remove heavy metals^5,6^.

To date, the effect of several fucoidan products from different algal species on immune activity has been reported. Fucoidan structures can vary from species to species and even according to the extraction method, and these differences may cause differences in biological activities such as immunological activities^7,8^. In the research of Lee et al.^9^, the anti-inflammatory effects of fucoidan extracted from *Ecklonia cava* algae were investigated in zebrafish as a model. The results showed that fucoidan extracted from *E. cava* has significant anti-inflammatory activity against tail amputation and inflammation^9^. Kang et al.^10^ reported the protective effect of polyphenol extract extracted from this alga against apoptosis and cell damage caused by ethanol and oxidative exposure in vitro and in vivo conditions^10^.

The study conducted by Abdel-Daim et al.^11^, investigated the impact of feeding Nile tilapia (*Oreochromis niloticus*) with aflatoxin and fucoidan extracted from *Laminaria japonica*. The findings showed that the group fed with aflatoxin had high levels of ALT, AST, ALP, cholesterol, urea, and creatinine, as well as significantly decreased amounts of total protein, glutathione peroxidase, superoxide dismutase, and catalase^11^. Conversely, the group fed with fucoidan showed a significant decrease in the activity levels of ALT, AST, and ALP enzymes, cholesterol, urea, and creatinine, while the levels of total protein, glutathione peroxidase, superoxide dismutase, and catalase were increased^11^. Seaweeds are considered a vast and renewable source of natural compounds, many of which have been found to possess bioactive properties that could be beneficial for various applications, including agriculture, aquaculture and food industry. In particular, seaweed-derived sulphated polysaccharides (SPs) have shown potential for their ability to enhance growth and improve immunity in animals. However, little is known about the potential of SPs to mitigate stress caused by environmental stressors, such as copper sulphate, in fish farms. Therefore, the aim of this study was to investigate the effects of oral consumption of SPs on mitigating the stress caused by copper sulphate in fish farms and to evaluate their potential as a dietary supplement for aquatic animals.

## Materials and methods

### Fish and treatment

Zebra fish (*Danio rerio*) were purchased from the breeding center of ornamental fish. Initially, the fish were cultivated for a period of two weeks to facilitate acclimatization and ensure their health. During this interval, they were provided with a standard commercial diet. After that SPs supplement diet was used for 8 weeks in 5 treatments (each treatment with 3 replicates) including negative control (N. Con.), positive control (P. Con.) fed without SPs, the group fed with 0.5%, 1% and 1.5% of SPs, then they were exposed to CuSO_4_ at the end of culture period for 1 week. After anaesthetization of the fish for gene expression studies and whole-body extract (WBE) assays, whole-body samples were dried with clean and sterilized cloths to remove excess water and then immediately immersed in liquid nitrogen. The WBE was extracted in phosphate buffer (pH 7.4) and supernatant was used for WBE assays and RNA was extracted using the Wizol extraction kit^12^. For histological studies, liver sampling was done. During the rearing period, water temperature, dissolved oxygen and total hardness were measured as 26 ± 1.5 °C, 6 ± 0.98 mg/L, and 470 ± 25 mg/L, respectively.

### Food preparation

The sulphated polysaccharide used in this study was extracted from *Sargassum tenerimum* algae using the method of Yang et al.^13^. The required amounts of SPs were weighed, dissolved in distilled water, and added to the diet as a spray. Daily feeding consisted of 3% of the biomass for the first 3 weeks and 5% of the biomass for the last 5 weeks of the culture period for each treatment.

### Exposure to copper sulfate

CuSO_4_ was purchased in powder form with Sigma brand. According to the water conditions of the reservoirs before the experiment, to determine the LC_50_, a pre-test was conducted, in which 60 fish were exposed to different concentrations of CuSO_4_ (0, 0.05, 0.1, 0.15, 0.2, 0.3, 0.4, and 0.5 mg/L) in triplicate. the LC_50_ value was calculated as 0.2 mg/L of CuSO_4_ in 96 h by examining the wide concentrations^14–16^ At the end of culture period with experimental diets, 60 fish were randomly selected for each treatment and then exposed to CuSO_4_. Concentration of CuSO_4_ in main study was used as $\frac{1}{2}$ of the LC_50_ for each tank (0.1 mg/L) for one-week exposure time.

### Whole-body extract assays

Total protein, albumin, cholesterol, glucose, ALP, ALT, and AST enzymes were measured by a commercial biochemistry kit using the spectrophotometric method (Biochrom, libra S12) based on the instructions inside the kits approved by Zeist Chem diagnostics, Iran. The amount of total immunoglobulin was calculated by dividing the protein concentration in the initial sample and the protein concentration after precipitation with polyethylene glycol^17^.

### Gene expression studies

After beating, the whole-body tissue samples were sonicated according to the instructions of the RNA extraction kit approved by Wizol kit. cDNA synthesis was performed using GENET BIO's cDNA synthesis Master-mix according to the kit's instructions. In order to measure the quality of extracted RNA, cDNA and confirm the quality of primers (the sequence and other specifications of the primers are shown in Table 1), normal PCR test along with agarose gel electrophoresis were performed at each stage, and finally, the samples were placed in the real-time PCR machine.

**Table 1:** Characteristics of the primers used in investigating the relative expression of lysozyme, Tnf-α and Il-1β genes in zebrafish.

Primer name	Primer sequence	Tm (°C)	Application	Accession number
Lyz q-PCRF	GGCAGTGGTGTTTTTGTGTC	58	Immune gene	NM_139180.1
Lyz q-PCRR	CGTAGTCCTTCCCCGTATCA			
Tnf-α q-PCRF	CTGCTTCACGCTCCATAAGA	58	Immune gene	AY427649.1
Tnf-α q-PCRR	CTGGTCCTGGTCATCTCTCC			
Il-1β q-PCRF	CGTCTCCACATCTCGTACTCA	58	Immune gene	AY340959.1
Il-1β q-PCRR	GTGTCTTTCCTGTCCATCTCC			
β-actin q-PCRF	AGCAGATGTGGATCAGCAAG	58	Housekeeping gene	NM_131031.1
β-actin q-PCRR	TACCTCCCTTTGCCAGTTTC			

### Histopathology

Liver tissue sections were prepared for cutting according to Santos et al.^18^ as dehydration, clarification, paraffinization and molding. 5 µm sections of liver tissue were prepared and pasted on a glass slide. Finally, histological studies and examination of possible liver tissue damage were done after staining with hematoxylin–eosin (H&E) by light microscope (Nikon ECLIPSE E100) with 100–400 times magnification^19,20^.

### Statistical studies, data analysis and ethical issues

To analyze the data, one-way analysis of variance was performed with SPSS_22_ software, and Duncan's test was used to compare the means. The difference between the means in different treatments was determined with a confidence level of 95%. The results of the relative gene expression were calculated using the 2^−ΔΔCt^ method in Excel software compared to the β-actin reference gene.

All experimental procedures were approved by the GUASNR ethics committee (Gorgan University of Agricultural Sciences and Natural Resources). It confirmed that all experiments were performed in accordance with relevant guidelines and regulations as described by the ARRIVE guidelines (PLoS Bio 8(6), e1000412, 2010).

### Ethical approval

All experimental procedures related to the fish were in accordance with ethical standards for ethical review of fish welfare in GUASNR (Gorgan University of Agricultural Sciences and Natural Resources).

The study is reported in accordance with ARRIVE (Animals in Research: Reporting In Vivo Experiments) guidelines. https://doi.org/10.1371/journal.pbio.1000412

## Results

### Survival

According to Table 2, the results of the study show the average percentage of mortality after exposure to copper sulfate in the negative control treatment was zero, in the positive control treatment it was 89.1%, in the treatments 0.5%, 1%, and 1.5% were 73.9%, 47.8%, and 39.1% respectively.

**Table 2:** Mortality rates at the end of CuSO_4_ exposure in zebrafish fed with diets containing different levels of SPs extracted from *S. tenerimum* algae.

	N. con	P. con	0/5	1	1/5
Weight (mgr)	502 ± 5^d^	499 ± 3^d^	542 ± 3^c^	590 ± 6^b^	598 ± 4^a^
Length (mm)	29 ± 1^a^	29 ± 2^a^	30 ± 2^a^	31 ± 3^a^	32 ± 2^a^
Mortality (%)	–	89/1	73/9	47/8	39/1

Different letters indicate significant differences between groups. Data showed as mean ± standard deviation.

### Glucose and cholesterol

**Figure 1:**
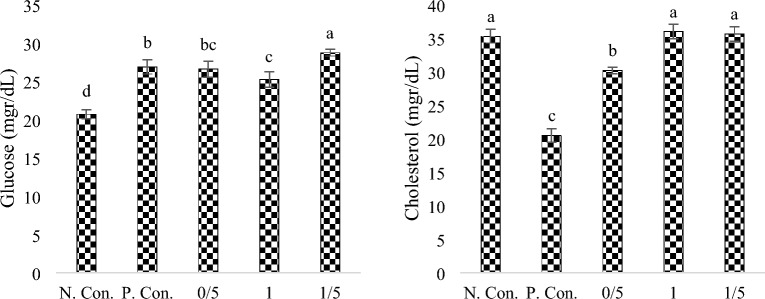
Glucose and Cholesterol in body extracts of zebra fish exposed under CuSO_4_ and fed with different levels of SPs extracted from *S. tenerimum* algae. Non-similar lowercase English letters on each column indicate a significant difference at the 0.05 level. Data are mean ± standard deviation.

The amount of glucose (Fig. 1) in the negative control treatment was significantly lower compared to other groups, and the highest level was in the group fed with a diet containing 1.5% SPs (sig < 0.05). Exposure to CuSO_4_ caused a significant decrease (sig < 0.05) in the blood cholesterol in the positive control and the group fed with 0.5% SPs, but no significant difference (sig < 0.05) was observed among other treatments (Fig. 1).

### Total protein, total immunoglobulin and albumin

**Figure 2:**
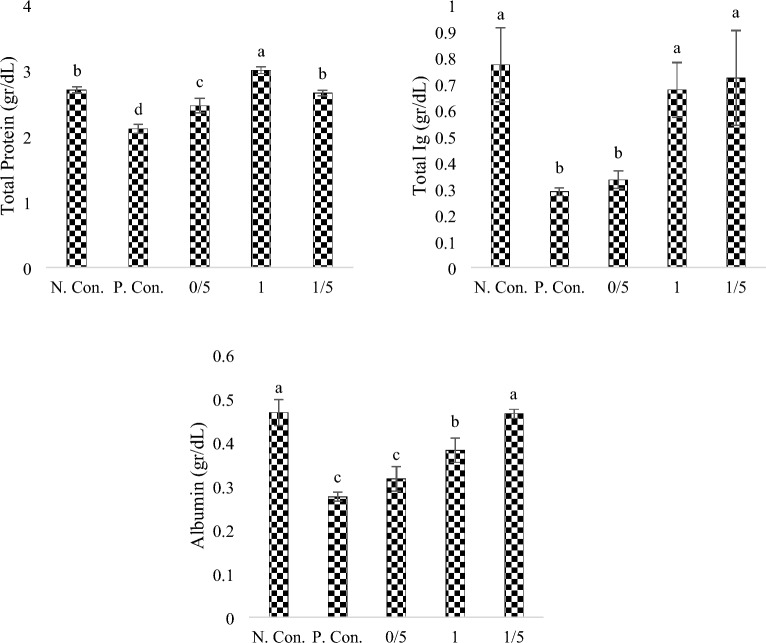
Total protein, Total Ig and Albumin levels in body extracts of zebra fish exposed under CuSO_4_ and fed with different levels of SPs extracted from *S. tenerimum* algae. Non-similar lowercase English letters on each column indicate a significant difference at the 0.05 level. Data are mean ± standard deviation.

According to Fig. 2, the highest and lowest significant levels (sig < 0.05) of total protein were measured in the group fed with 1% SPs and positive control group, respectively. No significant difference was observed between the negative control group and group fed with 1.5% SPs (sig < 0.05). The results of total immunoglobulin (Fig. 2) between the positive control group and the group fed with 0.5% SPs showed no significant difference (sig < 0.05), but total immunoglobulin level of these two groups were significantly lower compared to the other groups (sig > 0.05). In the groups under CuSO_4_ exposure (Fig. 2), the positive control group showed the lowest amount of albumin, and with the increase in the concentration of SPs, the level of albumin also significantly increased (sig < 0.05) so the highest albumin level was measured for the negative control group and the group fed with 1.5% SPs (sig < 0.05).

### ALT, AST and ALP enzymes

**Figure 3:**
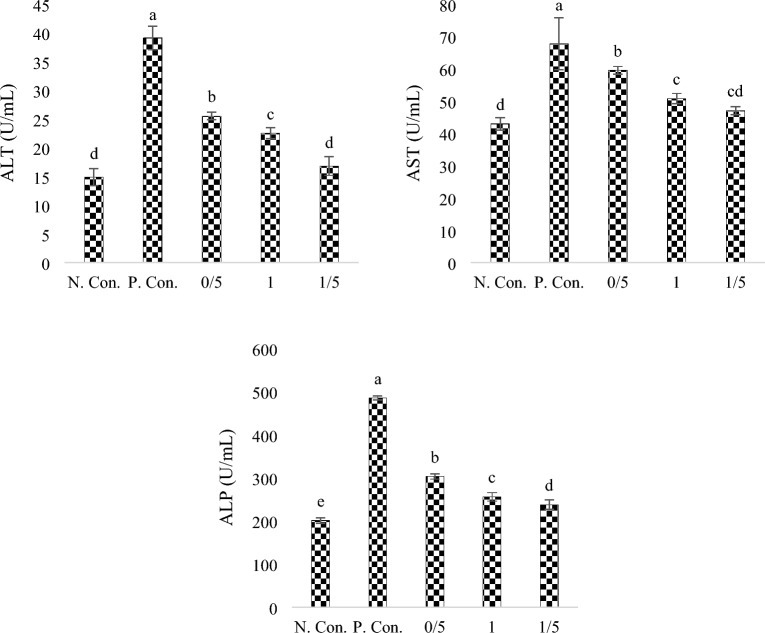
ALT, AST and ALP enzyme activity levels in body extracts of zebra fish exposed under CuSO_4_ and fed with different levels of SPs extracted from *S. tenerimum* algae. Non-similar lowercase English letters on each column indicate a significant difference at the 0.05 level. Data are mean ± standard deviation.

Amount of ALT enzyme (Fig. 3) show that in groups under CuSO_4_ exposure, it decreases significantly (sig < 0.05) with the increase in the concentration of SPs. The level of ALT enzyme in the negative control group and the 1.5% SPs group showed no significant difference (sig < 0.05). The comparison of AST enzyme level (Fig. 3) between the groups showed a significant difference in the positive control group and the negative control group (sig < 0.05). Also, a significant difference was seen in the negative and positive control groups with the 0.5% and 1% SPs groups (sig < 0.05). The level of ALP enzyme (Fig. 3) was the lowest in the negative control group (sig < 0.05) and the highest level was in the positive control group (sig < 0.05), which significantly decreased with the increase in the concentration of SPs (sig > 0.05).

### Expression of lysozyme, Tnf-α and Il-1β genes

**Figure 4:**
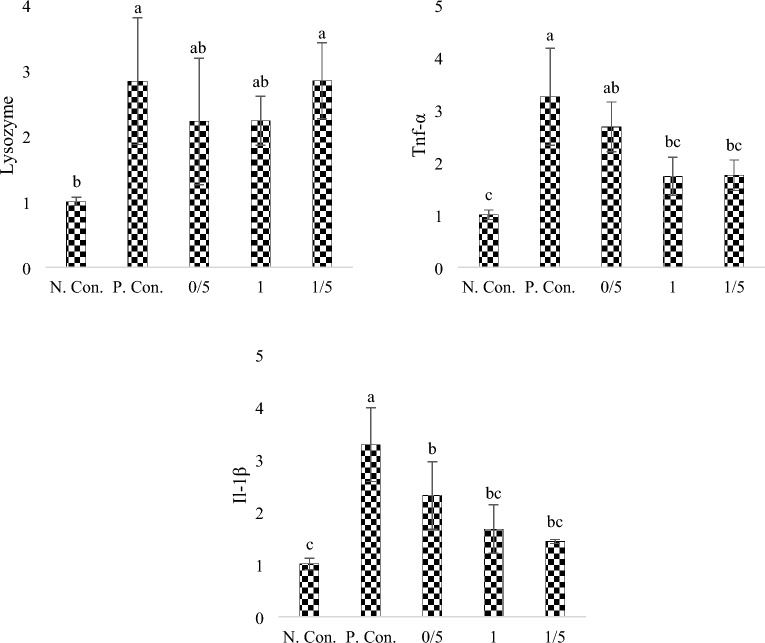
Relative expression level of lysozyme gene, tumor necrosis factor (Tnf-α) gene and interleukin gene (Il-1β) in zebra fish exposed under CuSO_4_ and fed with different levels of SPs extracted from *S*. *tenerimum* algae. Non-similar lowercase English letters on each column indicate a significant difference at the 0.05 level. Data are mean ± standard deviation.

According to Fig. 4, the highest relative expression level of lysozyme gene was in positive control and 1.5% SPs groups (sig < 0.05). The relative expression level of Tnf-α gene (Fig. 4) showed that the highest significant level (sig < 0.05) corresponds to the positive control group and the lowest significant level (sig < 0.05) corresponds to the 1 and 1.5% groups. According to Fig. 4, the highest significant level (sig < 0.05) of the relative expression of Il-1β gene found for the positive control group and the lowest level was also for the group 1 and 1.5% SPs (sig < 0.05).

### Liver histopathology

**Figure 5:**
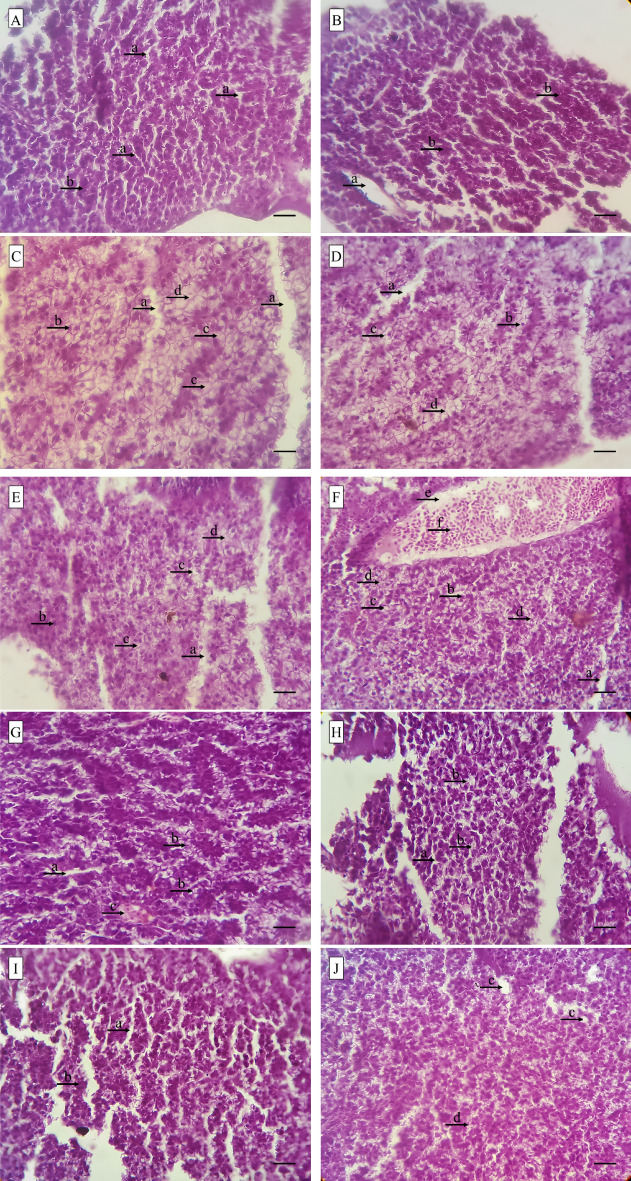
Liver tissue damage caused by CuSO_4_ exposure in zebra fish fed with different concentrations of SPs extracted from *S*. *tenerimum* algae. Figure (**A**) and (**B**) Liver tissue in negative control group (no exposure), normal bile duct (a), normal liver hepatocytes (b). Figure (**C**) and (**D**) Liver tissue in positive control group fish (CuSO_4_ exposure), bile duct (a), nucleus (b), intense vacuolation of liver hepatocytes (c), degeneration and necrosis in liver hepatocytes (d). Figure (**E**) and (**F**) Liver tissue of fish exposed to CuSO_4_ and fed with 0.5% SPs, normal bile duct (a), nucleus (b), vacuolation of liver hepatocytes (c), degeneration and the beginning of the process of necrosis in hepatocytes. Liver (d), central vein (e), blood cells (f). Figure (**G**) and (**H**) Liver tissue of fish exposed to CuSO_4_ and fed with 1% SPs, sinusoidal space (a), vacuolation of liver hepatocytes (b), vein with blood cells (c). Figure (**I**) and (**J**) Liver tissue of fish exposed to CuSO_4_ and fed with 1.5% SPs, normal bile duct (a), liver hepatocytes (b), sinusoidal space (c), beginning of vacuolation of liver hepatocytes (d).

Exposure to CuSO_4_ in zebrafish causes tissue damage in the liver. By increasing the concentration of SPs, the observed tissue damage was decreased (Table 3). The main damage observed in the liver tissue of treatments under CuSO_4_ exposure were liver hepatocyte vacuolation and necrosis in the positive control group and the 0.5% SPs groups (Fig. 5).

**Table 3:** Determination of liver lesions in zebra fish exposed under CuSO_4_ and fed with different levels of SPs.

Treatments	N. con	P. con	0/5	1	1/5
Liver lesions					
Ballooning degeneration	−	++++	+++	+	+
Necrosis	−	++++	+++	+	−
Destruction of sinusoidal space	−	+++	+	+	−
Hemosiderin deposition	−	−	−	−	−
Cirrhosis of liver	−	−	−	−	−
Cell vacuolation	−	++++	+++	+	+
Bleeding	−	+	−	−	−
Karyolysis	−	−	−	−	−
Enlargement of cell nucleus	−	+	+	−	−

No observed lesions (−),1 to 3 lesions (+), 3 to 5 lesions (++), 5 to 7 lesions (+++), more than 7 lesions (++++).

## Discussion and conclusion

Seaweeds are inexpensive sources of bioactive compounds that provide various properties such as growth stimulation, appetite stimulation, increased immunity and increased resistance to diseases in consumers^21^. In recent years, the use of bioactive compounds in various industries has gained special importance. However, the use of new compounds as nutritive-medicinal compounds requires a lot of research and research about the effect of these compounds on the physiological state and health of animals, so studying the various properties of bioactive compounds is very important.

### Survival rate and resistance to CuSO_4_ exposure

The resistance to CuSO_4_ exposure depends on factors such as water hardness, temperature, pH, species, the amount of manipulation, size and age, different biological stages and nutritional conditions. The occurrence of mass mortality in fish indicates the inability to adapt to environmental factors, and individual losses are often caused by the inability of individuals to adapt to the created conditions^22,23^. Survival percentage also indicates resistance to external factors and environmental exposures^24^.

This study shows the positive effect of SPs extracted from *S*. *tenerimum* algae on the tolerance of exposures conditions caused by the heavy metal copper in zebra fish. During this experiment, 89.1% of the positive control group died in 94 h under CuSO_4_ exposure. By increasing the concentration of SPs, the mortality trend showed a significant decrease, so that by adding 0.5% of SPs, the mortality rate was 73.9%, and by increasing the concentration of SPs to 1.5%, the mortality rate was decreased to 39.1%.

### Liver histopathology

In general, damage to the liver parenchyma causes an increase in serum transaminases^25^, and therefore an increase in AST, ALT and ALP enzymes is a sign of primary cell damage, which reflects the level of this enzyme in the blood serum. Heart, liver, kidney and spleen are among the primary organs that are affected by metabolic reactions caused by various substances. The liver, which is a key organ in the metabolism and detoxification of xenobiotic compounds^25^, may be vulnerable to various compounds. Liver is one of the main organs for plasma purification, homeostasis and removal of toxins and toxins from the body. Liver histological studies demonstrate the ability of SPs to increase resistance to the heavy metal copper^26–28^. The most liver damage was related to the positive control group that were fed only with the basic diet, and the amount of tissue damage decreased with the increase in the concentration of SPs. SPs has high antioxidant ability and strong chelation of heavy metals, which may have prevented it from increasing damage with its antioxidant properties when exposed to CuSO_4_^26–28^.

### Serological analysis

Given the involvement of body fluids in various metabolic processes, changes in their composition can serve as useful indicators of an animal's physiological status^29^. As such, serum evaluations are a critical diagnostic tool for assessing the health of living organisms^29^.

### Glucose and cholesterol

Blood glucose is a highly variable parameter that is strongly influenced by manipulative and environmental exposure such as nutritional status, seasonal changes, and sexual maturity^30^. Glucose level is widely used as an indicator of exposure to heavy metal in fish^31^. An increase in glucose concentration is a secondary response to stressors, so the amount of glucose increase indicates the body's response to metals exposure^32^. Cholesterol is a waxy substance with a lipid nature, which is necessary as a precursor for the synthesis of some hormones and vitamin D, which their major part is made by the liver and the intestinal wall.

In stress conditions, the blood glucose level can increase, according to the results of the positive control group, due to severe liver damage, glucose and cholesterol metabolism showed a significant decrease. The amount of exposure increased and cholesterol production decreased significantly. In the treatments receiving SPs due to its protective properties of liver cells^26–28^, less tissue damage was observed and the amount of exposure decreased, therefore, with increasing concentration of SPs, glucose and cholesterol levels approached normal levels, but did not decrease. The increase in glucose level compared to the negative control group may be due to two reasons, the sugary nature of SPs and the presence of CuSO_4_ exposure, as well as tissue damage and reduced liver metabolism^33,34^.

### Total protein, total immunoglobulin and albumin

A decrease in total protein, albumin and total immunoglobulin is a prominent feature of many diseases and may occur due to liver and kidney failure, decreased or loss of protein absorption^35–38^. In this study, by CuSO_4_ exposure and liver tissue damage, the amount of total protein, followed by the amount of albumin and total immunoglobulin in the positive control treatments and 0.5% group was significantly lower than other treatments. In groups under CuSO_4_ exposure and receiving 1 and 1.5 percent of SPs, the levels of total protein, albumin, and total immunoglobulin increased significantly. This may be due to the protective and antioxidant properties of this compound, which prevented the occurrence of impaired protein synthesis by the liver^26–28^.

### Activity of AST, ALT and ALP enzymes

ALT is a liver-specific enzyme that usually increases in response to liver tissue damage, whereas AST enzyme increases not only due to liver damage but also in cases of heart and muscle injuries. ALP is most active in an alkaline pH and is found in tissues such as the spleen, intestinal wall, thymus gland, and testicles, although its highest concentration is in the liver^35–40^. Normally, this enzyme's level rises in liver lesions and in cases of bile duct obstruction, cysts, and liver abscesses. In this study, adding SPs to the diet resulted in a significant decrease in the activity of AST, ALT, and ALP enzymes in all experimental treatments compared to the positive control group, indicating that SPs reduced the damage caused by CuSO_4_ and prevented tissue damage from spreading. The metal ion chelation and antioxidant properties of SPs are likely responsible for this effect^41,42^. As a result, the levels of AST, ALT, and ALP enzymes significantly decreased. The increase in the level of AST, ALT, and ALP enzymes in the positive control group suggests that CuSO_4_ induces stress conditions and impairs liver function, ultimately leading to mortality in this group.

### Expression of lysozyme, Tnf-α and Il-1β genes

Lysozyme hydrolyzes glycosidic bonds and is known as a lytic enzyme, and its activity level is usually considered as one of the parameters of innate immunity^23^. Il-1β is known as one of the pro-inflammatory factors that defends the body during the conflict with microbial pathogens and tissue damage by stimulating the increase of phagocyte activity, increasing the number of macrophages and the production of lysozyme^43^. Tnf-α also helps the innate defense system by stimulating cells to chemotaxis and phagocytosis in eliminating various pathogens^44^. Although an increase or decrease in the level of gene expression does not necessarily mean an increase or decrease in the production of the final product^45^, it can be said that in exposure induction, the level of transcription of the gene is changed to translate into a specific protein in order to carry out a specific reaction and overcome exposures conditions^46–48^. In this study, the relative expression level of Lysozyme gene in the treatments does not depend on the treatments, but it is possible that due to the CuSO_4_ exposure, the level of gene expression has increased in order to be able to cope with the invasion of pathogens. By dietary adding SPs, the living organism had a greater ability to tolerate exposures conditions and also has less liver damage. In this case, it may be due to the protective and antioxidant property of polysaccharide^41,42^, the expression level of Il-1β and Tnf-α genes had shown a significant decrease in doses of 1% and 1.5% of SPs to deal with inflammation and phagocytotic response.

## Conclusion

Further detailed studies are necessary to draw definitive conclusions regarding the effectiveness of SPs as a supplement for commercial, industrial, and pharmaceutical uses. However, in the present study, the observation of reduced tissue damage and a significant decrease in the activity level of ALT, AST, and ALP enzymes, which is supported by the results of histological studies, suggests that SPs, with their chelating and antioxidant properties, have the potential to protect against copper exposure and may serve as an effective supplement.

## Data Availability

The datasets generated and analyzed during the current study are not publicly available due to the funding responsibility but are available from the corresponding author on reasonable request.
