# Author Correction: Modulation of toxicity effects of CuSO_4_ by sulfated polysaccharides extracted from brown algae (*Sargassum tenerrimum*) in *Danio rerio* as a model

**DOI:** 10.1038/s41598-023-48934-4

**Published:** 2023-12-18

**Authors:** Ashkan Zargari, Mohammad Nejatian, Sepideh Abbaszadeh, Kambiz Jahanbin, Tahereh Bagheri, Aliakbar Hedayati, Monireh Sheykhi

**Affiliations:** 1 Department of Nutrition Science and Food Hygiene, Faculty of Health, Baqiyatallah University of Medical Sciences, Tehran, Iran; 2 Health Research Center, Life Style Institute, Baqiyatallah University of Medical Sciences, Tehran, Iran; 3 Faculty of Agricultural Engineering, Department of Food Science and Technology, Shahrood University of Technology, Shahrood, Iran; 4 Offshore Water Research Center (OWRC), Iranian Fisheries Science Research Institute, Agricultural Research, Education and Extension Organization (AREEO), Chabahar, Iran; 5 Faculty of Fisheries and Environmental Sciences, Gorgan University of Agricultural Sciences and Natural Resources, Gorgan, Iran; 6 Student Research Committee, Baqiyatallah University of Medical Sciences, Tehran, Iran

Correction to: *Scientific Reports* 10.1038/s41598-023-38549-0, published online 15 July 2023

In the original version of this Article, Ashkan Zargari was incorrectly listed as a corresponding author. The correct corresponding author for this Article is Mohammad Nejatian. Correspondence and request for materials should be addressed to mnejaatian@gmail.com.

In addition, Ashkan Zargari was incorrectly affiliated with ‘Faculty of Fisheries and Environmental Sciences, Gorgan University of Agricultural Sciences and Natural Resources, Gorgan, Iran’. The correct affiliation is listed below.

Department of Nutrition Science and Food Hygiene, Faculty of Health, Baqiyatallah University of Medical Sciences, Tehran, Iran.

The original Article has been corrected.

